# Identification and characterization of proteins that form the inner core *Ixodes scapularis* tick attachment cement layer

**DOI:** 10.1038/s41598-022-24881-4

**Published:** 2022-12-09

**Authors:** Albert Mulenga, Zeljko Radulovic, Lindsay Porter, Taylor Hollman Britten, Tae Kwon Kim, Lucas Tirloni, Alex Kiarie Gaithuma, Grace O. Adeniyi-Ipadeola, Jolene K. Dietrich, James J. Moresco, John R. Yates

**Affiliations:** 1 Department of Veterinary Pathobiology, College of Veterinary Medicine, Texas A&M University, College Station, TX USA; 2 Department of Molecular Medicine, The Scripps Research Institute, La Jolla, CA USA; 3 Mass Spectrometry Core, Salk Institute for Biological Studies, La Jolla, CA USA; 4 Present Address: Department of Biology, Stephen F. Austin State University, Nacogdoches, TX USA; 5 Present Address: Laboratory of Bacteriology, National Institute of Allergy and Infectious Diseases, Hamilton, MT USA; 6 Present Address: Department of Diagnostic Medicine/Pathobiology, College of Veterinary Medicine, Kansas State University, Manhattan, KS USA; 7 Present Address: Center for Genetics of Host Defense, UT Southwestern Medical Center, Dallas, TX USA; 8 Present Address: Department of Molecular Virology and Microbiology, Baylor College of Medicine, Houston, TX 77030 USA

**Keywords:** Molecular biology, Physiology

## Abstract

*Ixodes scapularis* long-term blood feeding behavior is facilitated by a tick secreted bio adhesive (tick cement) that attaches tick mouthparts to skin tissue and prevents the host from dislodging the attached tick. Understanding tick cement formation is highly sought after as its disruption will prevent tick feeding. This study describes proteins that form the inner core layer of *I. scapularis* tick cement as disrupting these proteins will likely stop formation of the outer cortical layer. The inner core cement layer completes formation by 24 h of tick attachment. Thus, we used laser-capture microdissection to isolate cement from cryosections of 6 h and 24 h tick attachment sites and to distinguish between early and late inner core cement proteins. LC–MS/MS analysis identified 138 tick cement proteins (TCPs) of which 37 and 35 were unique in cement of 6 and 24 h attached ticks respectively. We grouped TCPs in 14 functional categories: cuticular protein (16%), tick specific proteins of unknown function, cytoskeletal proteins, and enzymes (13% each), enzymes (10%), antioxidant, glycine rich, scaffolding, heat shock, histone, histamine binding, proteases and protease inhibitors, and miscellaneous (3–6% each). Gene ontology analysis confirm that TCPs are enriched for bio adhesive properties. Our data offer insights into tick cement bonding patterns and set the foundation for understanding the molecular basis of *I. scapularis* tick cement formation.

## Introduction

*Ixodes scapularis* is one the most medically important tick species as it transmits many human tick-borne disease (TBD) agents^1^. *I. scapularis* ticks were recognized as the principal transmission vector for 7 of the 16 human TBD agents reported in the United States of America including agents of Lyme disease, relapsing fever, human anaplasmosis, human babesiosis, and Powassan encephalitis virus^1^. Although there is considerable research effort toward developing vaccines against TBD agents, success is yet to come. In the absence of effective vaccines against TBD agents, disrupting tick feeding is the most reliable method to prevent infections with TBD agents and understanding tick feeding physiology is the critical first step^2^.

Like other hard ticks, *I. scapularis* is a long-term blood feeding ectoparasite. This feeding behavior is facilitated by a tick secreted bio adhesive (tick cement) that glues the tick mouthpart to host skin, stabilizes the feeding site, protect mouthparts from the host immune system, and above all protect the tick from being dislodged by the host^3^. Chemical analysis studies have shown that tick cement is mostly protein based followed by lipid and carbohydrates as reviewed^3^. Amino acid residue profiling have shown that tick cement proteins have high content of glycine and other hydrophobic amino acid residues^3^. On this basis, annotation of putative tick cement proteins has been based on abundance of glycine residues^2^. The limitation of these annotations is the lack of direct relationship of annotated tick cement proteins with tick cement formation.

Despite the importance of tick attachment cement, very little is known on proteins that form tick cement. To date, only two studies have identified and described proteins in *Amblyomma americanum* tick cement by LC–MS/MS analysis^4,5^. Tick cement has two layers, the inner core and the outer cortical. The rapidly hardening inner core layer start to be deposited within 5–30 min of the tick inserting the mouthparts into skin and completes formation by 24 h. The outer cortical layer that is laid around the inner core is deposited from 24 h of tick attachment and continues to harden through 96 h^3^. Here we focused on identifying proteins in the inner core layer as disrupting these will likely disrupt formation of the outer cortical. We have successfully used laser capture microdissection to isolate deposited cement from *I. scapularis* tick attachment sites in rabbit skin and LC–MS/MS analysis to identify proteins that form cement. While descriptive our data has provided insights into the molecular basis of tick cement formation, which is the critical first step toward disrupting tick cement as a target for tick control.

## Materials and methods

### Ethics statement

The use and care of experimental animals was done in accordance with ARRIVE (Animal Research: Reporting of In Vivo Experiments; https://arriveguidelines.org) guidelines. All experiments were performed according to the animal use protocol approved by Texas A&M University (TAMU) Institutional Animal Care and Use Committee (IACUC) (2014-0311) that meets all federal requirements, as defined in the Animal Welfare Act (AWA), the Public Health Service Policy (PHS), and the Humane Care and Use of Laboratory Animals. Rabbits were sedated and then euthanized with sodium pentobarbital as approved by TAMU IACUC.

### Tick feeding and attachment site biopsy

Adult *I. scapularis* ticks used in this study were obtained from Biodefense and Emerging Infections Research (BEI) Resources Repository. Ticks were fed on New Zealand white rabbits according to TAMU IACUC as described^6^. To prevent ticks from accessing the ear canal, the feeding area was restricted to the top of the rabbit ear using the 2-in. cotton stockinet tick containment cell. The tick containment cell was glued onto skin using the Kamar adhesive (Kamar Products Inc., Zionsville, IN, USA). Prior to feeding, ticks were pre-mated. To mate, ticks were placed into a petri dish on ice, and we visually monitored for paired male and female ticks, which were subsequently placed into a separate container to complete mating. We have observed that, there is synchronized attachment for pre-mated female ticks, which was important in this study, to sample ticks at similar phase in tick cement deposition process. Tick attachment was validated at 3 h after placing ticks into containment cells, and unattached ticks were removed.

The objective of this study was to identify proteins that form the inner layer of the tick cement cone that is deposited around tick mouthparts within 24 h of tick attachment because disrupting these proteins may interfere with the stability of the tick cement cone. The tick cement cone is comprised of the rapidly and slow hardening inner and outer layers respectively^3^. The inner layer starts to form within 5–30 min of the tick inserting its hypostome into the skin and completes hardening within 24 h of attachment while the outer layer deposition commences from 24 through 96 h of attachment^3^. Another consideration in our approach was to distinguish between early and late-stage inner layer tick cement proteins. Thus, we made skin punch biopsies of attachment sites for ticks that attached for 6 h and 24 h to segregate early and late-stage proteins that form the inner layer of the tick cement cone.

To make attachment biopsies, rabbits were sedated, and ticks were trimmed off above the mouthparts using scissors. This was done to prevent ticks from abandoning the feeding site after biopsy. The tick mouthparts were used as guide to locate the feeding site on biopsies. Subsequently, rabbits were sacrificed by pentobarbital sodium overdose according to the approved TAMU IACUC. Immediately after, skin punch biopsies of tick attachment sites were taken using 6 mm diameter biopsy puncher (Integra Life Sciences, Princeton, NJ, USA). Biopsies were placed into optimal cutting temperature compound (Life Technologies, Austin, TX, USA) and immediately snap frozen on dry ice and later transferred to −80 °C until processed.

### Cryo-sectioning of tick attachment site biopsies

The TAMU, College of Veterinary Medicine & Biomedical Science histology core performed transverse cryo-sectioning (10 μm) of tick attachment biopsies using Leica CM1850 UV Cryostat (Deer Park, IL). The rationale for transverse sectioning is that the location of tick mouthparts in the section guided the location of the tick attachment cement. Sectioning of biopsies began from the tick side and proceeded along the length of the mouthpart and finishing at the tick-feeding cavity where tick mouthparts were absent. Cryosections were placed onto PEN membrane glass slides (Life Technologies, Austin, TX, USA) which allowed for efficient recovery of dissected sections onto sample caps. Slides were stored in a slide box with a desiccant at – 80 °C until laser capture microdissection.

### Laser capture microdissection

Prior to laser capture microdissection, cryosections were stained using the Arcturus HistoGene LCM Frozen Section Staining Kit (Thermo Scientific). Slides from were thawed on paper towel for under a minute. This is a critical step, as our preliminary work showed that tissues detached if slides were not completely thawed. Thawed sections were fixed in 70% ethanol for 30 s, rinsed in deionized water, and incubated in staining solution for 20 s: prolonged exposure can damage sections. Excess stain was removed by rinsing in deionized water followed by 70, 95, and 100% ethanol washes for 30 s each. Subsequently, slides were cleared in xylene and then dried on a paper towel in a fume hood. Stained slides were kept in a desiccator until the micro-dissection procedure.

The Arcturus Pixcell II LC Microscope (Leica Microsystems, Deerfield, IL, USA) was used to micro dissect tick cement. The laser capture microdissection was done in two steps. Under a dissection scope the tick cement cone was identified as the lighter-staining region between the darker, chitinous tick mouthparts and the darker-staining adjacent skin tissue. The tick cement cone (or target area) perimeter was then cut around with an ultraviolet laser. Multiple dissected tick cement cones were recovered onto sample caps and processed for protein extraction. Total proteins were extracted into 50 μL denaturing buffer (6 M urea, 0.5 M NaCl, 50 mM Imidazole, 20 mM Tris–HCl, pH7.9) overnight at 4 °C. To prevent diluting samples, the same extraction buffer was used to extract from several sample caps. In preliminary studies we determined that 50uL was the minimum volume required to cover the sample recovery cap surface. Quantification of total protein was done using the enhanced option after appropriate dilutions in the Pierce BCA Protein Assay Kit (ThermoFisher, Waltham, MA, USA).

In our preliminary analysis we observed that dissected cement contained pieces of the tick mouthpart. Thus, we extracted proteins of unfed tick mouthparts to distinguish tick mouthpart proteins that might have no role in tick cement formation. To extract tick mouthpart proteins, unfed tick mouthparts were cut off (n = 50 ticks) and chopped up into small pieces using scalpel. Subsequently, proteins were extracted into 6 M urea denaturing buffer (as above) with sonication using a CL18 Dismembranator (ThermoFisher).

### LC–MS/MS analysis

Identification of proteins was done as previously published by our lab^2,6,7^. Briefly, total tick cement protein extracts (n = 3 μg × 3) were analyzed by LC–MS/MS at the Scripps Institute. Protein extracts were digested in solution using trypsin and analyzed using the nanoflow LC–MS/MS analysis on the Easy NanoLC II and a QExactive mass spectrometer (Thermo Scientific). To identify tick cement proteins, MS/MS tandem mass spectra was queried against the database of non-redundant *Ixodes* tick and rabbit protein sequences using the Integrated Proteomics Pipeline—IP2. Tick and rabbit protein sequences were downloaded from public databases (NCBI and Uniprot). Each protein entry in the database was auto reversed by the software for false discovery assessment, which was allowed at 1% or less. Proteins for which at least two peptides appeared in two of the three independent runs were retained.

### Functional annotation and in silico physicochemical analysis

Tick cement proteins (TCPs) were mapped into a hyperlinked excel spreadsheet. Functional annotation was done by querying tick cement proteins (TCPs) against different databases. Signal peptide, transmembrane domains, furin cleavage sites, and glycosylation sites and other post translational modifications were determined with software from the Center for Biological Sequence Analysis (https://www.cbs.dtu.dk/services/). The automated annotation of the proteins was based on matches to databases, including CDD, PFAM, and SMART, TickSialoFam (TSF), and molecular function gene ontology (GO) terms at the geneontology.org server^8^. We previously described 226 proteins in tick cement that was recovered from manually detached *A. americanum*^4^. Thus, to get insights into cement proteins that were conserved between *I. scapularis* and *A. americanum,* we searched TCPs here against *A. americanum* cement proteins^4^. Likewise, we searched TCPs in this study against *I. scapularis* tick saliva proteins that are secreted by 24 to 120 h fed ticks^6^ to identify TCPs that are also injected into tick saliva.

To determine differential enrichment, normalized spectral abundance factors were loaded into iDEP (integrated Differential Expression and Pathway analysis), available at http://ge-lab.org/idep/ to generate heatmaps. Hierarchical clustering of genes based on expression pattern across all sample groups was done using k-means clustering and visualized in a heatmap. For TCPs, classification of enriched protein families in each cluster was done on PANTHER (geneontology.org), based on enriched gene ontology (GO) terms where over-representation test was determined using the Fisher's exact test (*P* < 0.05) and Bonferroni correction for multiple testing^8^.

To get insight into conserved amino acid motifs, sequences were aligned in MacVector software (MacVector Inc., Apex, North Carolina, USA) and if applicable, aligned conserved motifs were visualized at Weblogo version 2.8.2 (https://weblogo.berkeley.edu/) ^9^. Amino acid composition was enumerated on bioinformatics.org server (https://www.bioinformatics.org) and classified as hydrophobic, hydrophilic, or charged as previously defined^10^. Amino acid residue composition was reported as the average count for proteins identified in cement of ticks that attached for 6 h and 24 h and those that were shared.

## Results and discussion

### Identification of proteins in inner core *I. scapularis* tick cement

**Figure 1:**
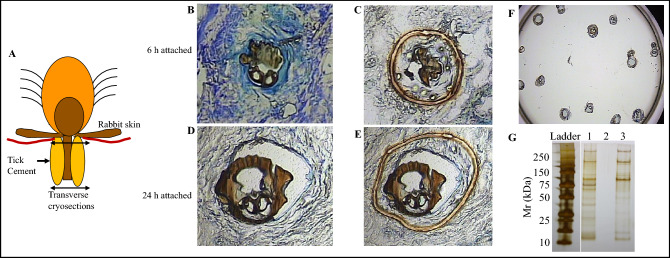
Microdissecting the inner core tick cement layer of *Ixodes scapularis* tick attachment cement. Adult female ticks were attached on rabbits for 6 and 24 h on rabbits. Skin punch biopsies of tick attachment sites were harvested using the 6 mm diameter biopsy puncher and placed into optimal cutting temperature compound (OCT), snap frozen on dry ice and stored at −80 °C until processed. Tick attachment biopsies were longitudinally cry sectioned (**A**), placed on PEN membrane slides and stained using the Arcturus HistoGene LCM Frozen Section Staining Kit. Tick attachment cement was located by position of mouthpart and lighter stain retention than adjacent skin tissue (**B,D**). Tick cement was dissected using the ultraviolet laser on Arcturus Pixcell II laser capture microscope (**C,E**). Dissected tick cement was recovered onto sample caps (**F**) and total proteins extracted overnight into 6 M urea buffer (**G**). Lanes 1 and 3 in (**G**) = protein extracts in cement of 6 and 24 h attached ticks, and lane 2 is an empty lane.

We for the first time successfully used Laser Capture Microdissection (LCM) to isolate the inner core layer of *I. scapularis* tick attachment cement (Fig. 1). The inner core tick cement completes forming by 24 h of tick attachment^3^. Thus, to attempt at identifying early and late-stage inner core tick cement proteins (TCPs), we made skin punch biopsies of 6 h and 24 h attachment sites. Subsequently, tick attachment site biopsies were transversely cryo-sectioned (Fig. 1A) and the tick cement in cryosections was located by light staining (compared to dark staining rabbit tissue) and proximity to the mouthpart (Fig. 1B,D). Tick cement was dissected out using LCM (Fig. 1C,E), recovered onto sample caps (Fig. 1F), and total protein was extracted into urea buffer overnight at 4 °C (Fig. 1G).

LC–MS/MS analysis approach^2,6,7^ was used to identify TCPs and rabbit proteins associated with tick cement (Supplemental Table [ST] 1 and ST2A). Searching extracted tandem mass spectra against the non-redundant database of *I. scapularis* and rabbit proteins identified a combined 660 and 714 tick and rabbit proteins respectively (ST1 and ST2A). Of the 660 tick proteins, 138 were in cement and the rest in tick mouthpart (ST1A). The 138 TCPs include 37 and 35 that were exclusively found in cement of 6 h and 24 h attached ticks (ST1A). It is notable that 47 of the 138 tick cement proteins were exclusively found in cement and not in the mouthpart (ST1A). The 47 proteins include 23 and 16 that were respectively identified in cement of 6 and 24 h attached ticks and the rest were shared (ST1A).

**Figure 2:**
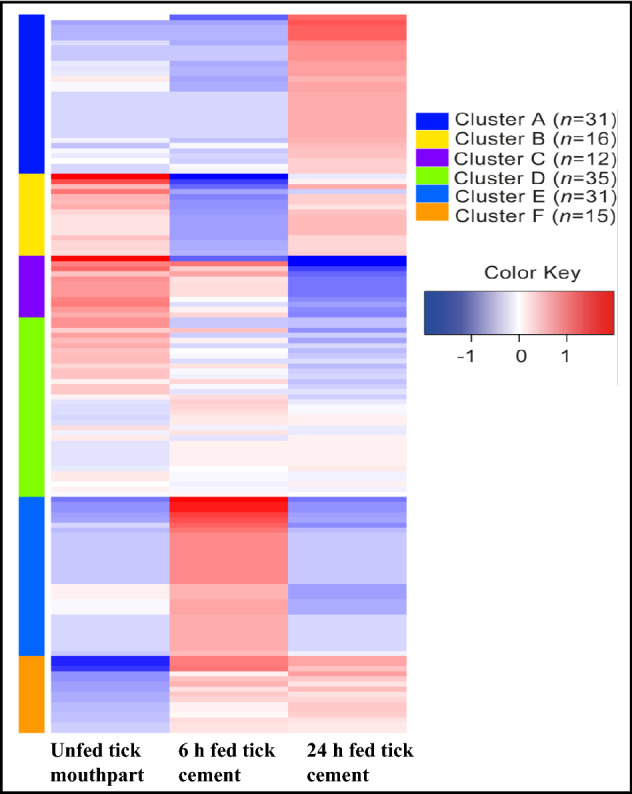
Relative abundance of tick cement proteins in cement of 6 and 24 h attached ticks and in mouthparts of unfed ticks. Normalized spectral abundance factor were loaded into iDEP (integrated Differential Expression and Pathway analysis; http://ge-lab.org/idep/) to generate the heat map. Accession numbers of tick cement proteins in different clusters of the heatmap are listed in supplemental Table 1B.

It is important to note that dissected tick cement contained pieces of the tick mouthpart (Fig. 1C,E), and there is a possibility that some of the TCPs could be mouthpart proteins not related to tick cement formation. However, we are encouraged by the fact that nearly 68% (93/138) in clusters A, B, E, and F were highly abundant in cement of 6 and 24 h attached ticks (Fig. 2). NCBI accession numbers of TCPs in different clusters (Fig. 2) are provided (ST1B).

### Annotation and Gene Ontology analysis

**Figure 3:**
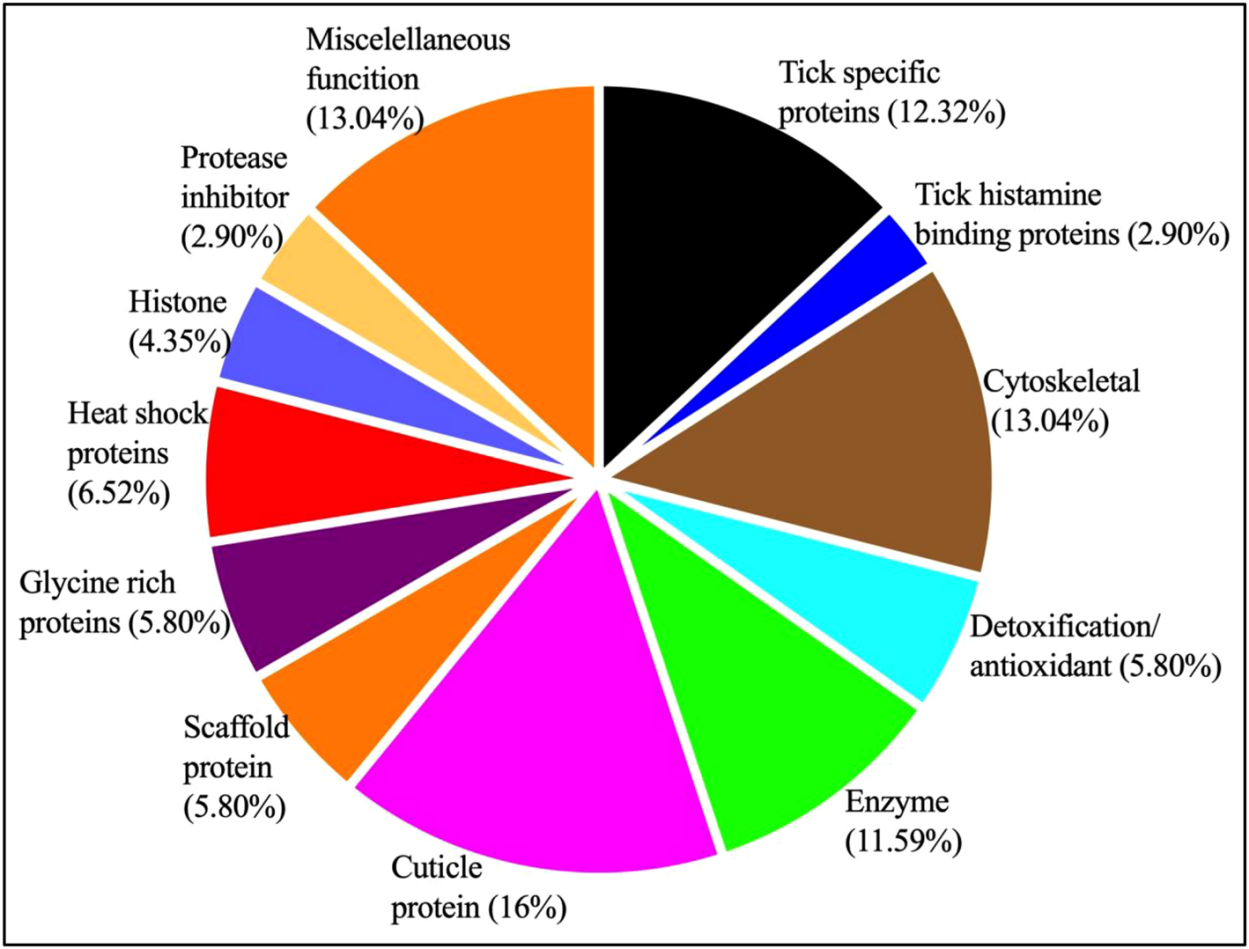
Protein functional categories in the inner core layer of *I. scapularis* tick attachment cement. Functional categories were determined based on matches to annotated proteins and conserved domain databases as summarized in supplemental Table 1C. Total protein counts in each functional category were plotted in PRISM 9.

Comprehensive annotation of tick cement proteins (TCPs) was accomplished by searching against multiple databases (ST1C). Based on this comprehensive annotation, we grouped TCPs into 14 functional categories: cuticle proteins (16%), cytoskeletal (13%), tick specific proteins of unknown function and enzymes both at 12%, detoxification/antioxidant, glycine rich, heat shock, and histone each at 4–7%. Others at under 3% include tick histamine binding and protease inhibitors and several classified under miscellaneous function accounting for 13% proteins (Fig. 3 and ST1C).

We previously described 226 proteins in cement that was recovered from mouthparts of manually detached *A. americanum* ticks^4^. It is interesting to note that 78 of 138 TCPs here are 40–100% identical to *A. americanum* TCPs (ST1C). Glycine rich proteins (GRP) are considered important in tick cement formation^3^. Thus, it is noteworthy that 6 of 8 GRPs (EEC15718.1, EEC15723.1, EEC15720.1, AAY66550.1, EEC00903.1, and EEC14464.1) here are conserved in *A. americanum* cement (ST1C).

Querying against tick saliva proteins that are secreted by adult *I. scapularis* identified 31 of the 138 TCPs that are also secreted during the 24–120 feeding stages^6^. We speculate that these proteins regulate functions beyond tick cement formation (ST1D). The 31 proteins found in both tick cement and saliva include cuticular proteins (EEC17151.1, EEC07075.1, EEC04237.1, EEC03471.1, and EEC08503.1), cytoskeletal (EEC08136.1), detoxification/antioxidant (EEC05916.1, AAY66786.1, EEC14604.1), enzyme (EEC17118.1, EEC13429.1), and GRP (EEC14464.1, EEC14470.1). Others, include heat shock proteins (EEC05056.1, EEC18473.1), miscellaneous function (EEC13579.1, EEC13271.1, AAY66716.1, EEC20127.1, AAV80782.1, EEC14178.1, EEC09465.1), protease inhibitor (AAY66685.1, EEC07262.1, EEC17564.1), scaffold protein (EEC14106.1, EEC11563.1), tick histamine binding proteins (AAM93664.1, AAY66819.1), and tick specific protein (EEC03925.1, EEC02397.1, EEC13528.1, AAY66605.1).

To gain further insights into function, TCP clusters (Fig. 2) were queried against molecular function GO terms in Panther^8^. Not surprisingly, TCPs in this study are enriched for functional properties that will promote tick cement formation including binding of bio molecules such as protein, carbohydrates, and nucleic acids, protein heterodimerization, and structural constituent activities (Table 1). Please note that the full list of molecular function Go terms is provided and IDs of proteins that mapped GO terms are listed (ST1E and ST1F). We have also provided but not discussed in detail proteins tick mouthpart only (ST1G).

**Table 1:** Molecular function gene ontology (GO) terms enriched in tick cement.

Cluster	GO molecular function complete	All *Ixodes* Proteins with this GO term	Mapped proteins	Expected p-value	Fold enrichment	Raw P-value	FDR (< 0.05)
A	**Protein heterodimerization activity**	33	3	0.04	71.58	1.26E−05	1.72E−02
	Protein binding	1081	9	1.37	6.56	4.52E−06	1.23E−02
	Binding	5333	17	6.77	2.51	2.96E−05	2.69E−02
	**Unfolded protein binding**	58	3	0.07	40.73	6.21E−05	4.24E−02
B	**Misfolded protein binding**	14	4	0.01	> 100	4.15E−10	1.13E−06
	**Protein folding chaperone**	17	4	0.01	> 100	8.11E−10	1.11E−06
	**Heat shock protein binding**	29	4	0.02	> 100	5.52E−09	5.02E−06
	**Unfolded protein binding**	58	4	0.04	> 100	7.44E−08	5.08E−05
	**ATP hydrolysis activity**	96	4	0.07	60.93	5.15E−07	2.81E−04
	ATP-dependent activity	426	5	0.29	17.16	6.89E−06	2.35E−03
	Nucleoside-triphosphatase activity	262	4	0.18	22.33	2.51E−05	6.85E−03
	Pyrophosphatase activity	294	4	0.2	19.9	3.91E−05	9.72E−03
	Hydrolase activity, acting on acid anhydrides, in phosphorus-containing anhydrides	311	4	0.21	18.81	4.86E−05	1.11E−02
	Hydrolase activity, acting on acid anhydrides	311	4	0.21	18.81	4.86E−05	1.02E−02
	**2-Alkenal reductase [NAD(P)+] activity**	141	4	0.1	41.49	2.28E−06	1.04E−03
	Oxidoreductase activity, acting on the CH-CH group of donors, NAD or NADP as acceptor	167	4	0.11	35.03	4.39E−06	1.71E−03
	Oxidoreductase activity, acting on the CH-CH group of donors	193	4	0.13	30.31	7.70E−06	2.34E−03
	**Structural constituent of cuticle**	109	3	0.07	40.25	5.54E−05	1.08E−02
	**ATP binding**	883	5	0.6	8.28	2.18E−04	3.72E−02
	Adenyl ribonucleotide binding	904	5	0.62	8.09	2.43E−04	3.91E−02
	Carbohydrate derivative binding	1238	6	0.85	7.09	9.74E−05	1.77E−02
	Adenyl nucleotide binding	909	5	0.62	8.04	2.50E−04	3.79E−02
C	**Structural constituent of cytoskeleton**	22	3	0.01	> 100	1.92E−07	5.23E−04
D	**Structural constituent of cytoskeleton**	22	10	0.03	> 100	3.52E−22	9.60E−19
	Structural molecule activity	323	15	0.46	32.78	8.34E−20	1.14E−16
	**GTPase activity**	164	11	0.23	47.35	3.86E−16	1.76E−13
	Nucleoside-triphosphatase activity	262	11	0.37	29.64	5.34E−14	2.08E−11
	Pyrophosphatase activity	294	11	0.42	26.41	1.80E−13	6.14E−11
	Hydrolase activity, acting on acid anhydrides, in phosphorus-containing anhydrides	311	11	0.44	24.97	3.25E−13	9.87E−11
	Hydrolase activity, acting on acid anhydrides	311	11	0.44	24.97	3.25E−13	8.88E−11
	Hydrolase activity	2457	12	3.48	3.45	6.50E−05	6.34E−03
	**GTP binding**	194	12	0.27	43.67	3.43E−17	3.12E−14
	Purine ribonucleoside triphosphate binding	1072	15	1.52	9.88	2.57E−12	5.41E−10
	Nucleoside phosphate binding	1300	16	1.84	8.69	2.33E−12	5.29E−10
	Organic cyclic compound binding	3209	17	4.55	3.74	1.64E−07	2.13E−05
	Binding	5333	17	7.55	2.25	3.67E−04	3.46E−02
	Heterocyclic compound binding	3189	17	4.52	3.76	1.49E−07	2.04E−05
	Anion binding	1296	15	1.84	8.17	3.74E−11	5.37E−09
	Ion binding	2775	15	3.93	3.82	1.15E−06	1.25E−04
	Guanyl ribonucleotide binding	200	12	0.28	42.36	4.86E−17	3.32E−14
	Purine ribonucleotide binding	1097	15	1.55	9.65	3.57E−12	6.96E−10
	Purine nucleotide binding	1111	15	1.57	9.53	4.27E−12	7.77E−10
	Nucleotide binding	1300	16	1.84	8.69	2.33E−12	5.77E−10
	Small molecule binding	1402	16	1.99	8.06	7.24E−12	1.16E−09
	Ribonucleotide binding	1112	15	1.58	9.52	4.32E−12	7.38E−10
	Carbohydrate derivative binding	1238	15	1.75	8.55	1.97E−11	2.98E−09
	Guanyl nucleotide binding	208	12	0.29	40.73	7.62E−17	4.16E−14
	**Structural constituent of chitin-based larval cuticle**	101	5	0.14	34.95	3.61E−07	4.48E−05
	Structural constituent of chitin-based cuticle	101	5	0.14	34.95	3.61E−07	4.29E−05
	Structural constituent of cuticle	109	5	0.15	32.38	5.19E−07	5.91E−05
E	**Structural constituent of cuticle**	109	4	0.14	28.9	1.19E−05	3.25E−02
F	**Protein tag**	7	2	0	> 100	1.13E−05	3.08E−02
	**Structural constituent of cuticle**	109	3	0.06	46.96	3.38E−05	3.07E−02
	Structural molecule activity	323	4	0.19	21.13	2.85E−05	3.89E−02

We would like to advise the reader to interpret our data on rabbit proteins that were associated with tick cement (ST2A) with caution as we did not sequence non-tick infested rabbit skin for control. However, it is notable that expression of rabbit proteins was apparently not random. The heatmap (supplemental (S) Fig. 1) show that the top 500 most variable rabbit proteins segregated into five groups: cluster A (CA: highly expressed in tick mouthparts than rabbit skin), CB and CC (highly expressed skin around 24 h and 6 h cement), CD and CE (expressed at similar levels in skin around 24 and 6 h cement). We speculate that CA rabbit proteins (S Fig. 1) are housekeeping proteins that are conserved in ticks and could play roles in cement formation. Enriched pathway analysis (ST2B) suggests that some of the rabbit proteins in this study represent the rabbit’s response to tick feeding style of creating a wound and then sucking blood that bleeds into the feeding site. For instance, enriched pathways include immune response, antimicrobial activity, and keratinocytes regeneration that could promote tissue repair. Please also note that accession numbers rabbit that mapped to all pathways, molecular and biological function pathways are provided (ST2C, ST2D, and ST2E). In subsequent sections, we have discussed TCP functional categories.

### Cuticle proteins

**Figure 4:**
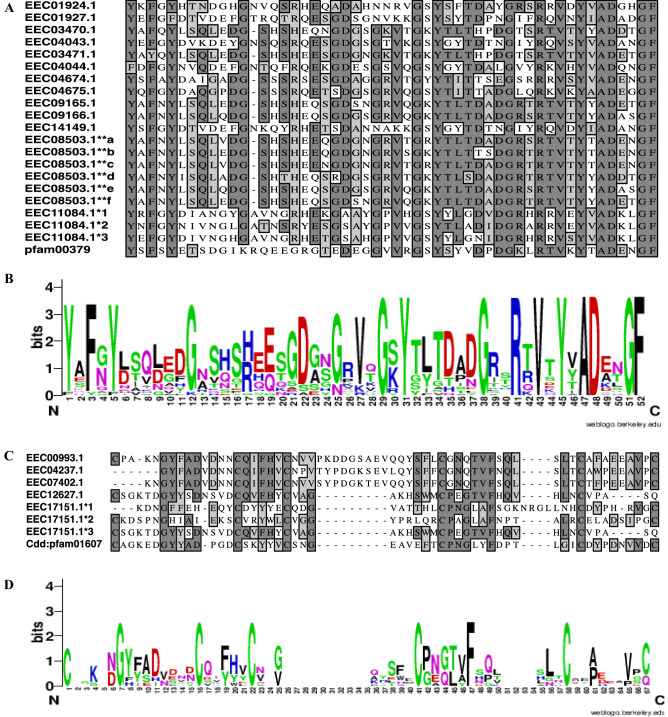
Sequence Logo for putative binding domains of chitin (pfam00379; Fig. 3A) and carbohydrate (pfam00167; Fig. 3C) in tick cement cuticle proteins. The pfam00379 and pfam00167 motifs as identified on the NCBI were aligned in MacVector (Fig. 3A,C) and sequence logos (Fig. 3B,D) visualized on the WebLogo server (https://weblogo.berkeley.edu/). 1**^a–f^ and 1*^1–3^ = pfam motifs in EEC08503 and EEC11084.1 and pfam01607 in EEC17151.1.

We identified 22 cuticle proteins (CPs) of which 5 (EEC12627.1, EEC11084.1, EEC17151.1, EEC09495.1, and EEC11064.1) and 3 (EEC00993.1, EEC07075.1, and EEC07402.1) in cement of 6 h and 24 h attached ticks, and 14 (EEC04237.1, EEC01924.1, EEC01927.1, EEC01928.1, EEC03470.1, EEC03471.1, EEC04043.1, EEC04044.1, EEC04674.1, EEC04675.1, EEC08503.1, EEC09165.1, EEC09166.1, and EEC14149.1) shared (ST1A). There are 12 CP families based on consensus motifs^11^. On this basis CPs in this study belong to CPR and CPAP (Cuticular Proteins Analogous to Peritrophins motif) families. The CPR and CPAP families are respectively characterized by pfam00379 (Rebers and Riddiford [RR] motif)^12^ and pfam01607 (or peritrophin-A domain) chitin/carbohydrate binding motifs^13^. The CPR and CPAP families are also respectively classified as chitin binding superfamily 4 (chitin_bind_4; cl02854) and carbohydrate-binding module (CBM_14; cl02629) on NCBI CDD database (ST1C). Multisequence alignment and sequence Logos show that both pfam00379 (Fig. 4A,B) and pfam01607 (Fig. 4C,D) are conserved in tick CPs^12,13^.

Most CPs have a single pfam00379 domain except for EEC08503.1 (or XP_029841824.1) and EEC11084.1 with multiple pfam00379 motifs and other amino acid repeat motifs (Supplemental Figure [SF] 1). Cuticle protein “XP_029841824.1” has six pfam00379 motifs flanked by “R-G-F-G-G-A-G-G-E-V-Y-A-P-I-P” repeats of unknown function (SF2A). Likewise, EEC11084.1 has three pfam00379 and two repeats of “R-A-V-V-K-T-N-E-P-G-T-K-[T/S]-S-[L/Y]-P-A-A-[A/P/S]-[P/A]” and a glycine rich c-terminal domain (SF2B). Of note, EEC04675.1 (or XP_029841826.1) has a single pfam00379 motif that is flanked by amino and c-terminus glycine rich domains (SF2C). The glycine rich repeats “H-H-G-G-G-G-F-G-G-G-G-L-G-G-H-H-G-G-G-G-F; G-G-G-G-L; G-G-G-G-R; G-G-Y; G-G-F, G-G-A, and G-G-G-G-G-Y” in EEC04675.1 have been described in elastomeric proteins^14^. Likewise, EEC12627.1 has a single pfam01607 motif flanked by glutamine (Q) rich domains at both the amino and c-terminal ends (SF2D). Poly Q domains have been shown to contribute to elastomeric protein properties^14^.

Although functional data are required, indirect evidence indicates that chitin binding is important to marine mussel bio adhesive adhesion to surfaces^15^ suggesting that some of the CPs in this study could bind chitin and promote tick cement adhesion to skin tissue surfaces. It is also possible for CPs to promote formation of the tick cement complex via glycosidic covalent bonds^16^ between /chitin bound carbohydrates bound on CPs.

### Scaffolding proteins (SP)

SPs bind other proteins to form an orderly functional complex^17^, which will promote tick cement formation. We identified 8 SPs in cement of 6 h (EEC10156.1, EEC12044.1, and EEC11563.1) and 24 h (EEC18671.1, EEC14737.1, EEC09647.1, and EEC17727.1) attached ticks, and shared (EEC14106.1). EEC10156.1 is a LIM domain protein, and its nomenclature is based on the on the occurrence of a double zinc finger motif “C-X_2_-C-X_17-19_-H-X_2_-C-X2-C-X2-C-X_15-19_-C” in 3 genes: *Caenorhabditis elegans lin-11,* rat insulin gene “*Isl-1,* and *C. elegans mec-3* (hence—LIM)^18^. EEC10156.1 is partial, and one motif is confirmed. Similarly, EEC14737.1 has a double zinc-finger domain (not shown) and might serve as an adaptor to other proteins. Putative makorin protein (EEC18671.1) contains a Really Interesting New Gene finger (RING) and U-box domains that contribute to large protein complex formation^19^. EEC12044.1 and EEC09647.1 have the Src homology 3 (SH3) domain which act as an adaptor for other proteins to form interactive complexes^20^. In addition to the SH3 domain, EEC09647.1 has an ankyrin repeat, which also promote protein–protein interactions^21^. EEC17727.1 contains the WD40 domain. The WD 40 domain protein is characterized by an 11–24 amino acid residue long glycine (G) and histidine (H) dipeptide at the amino-terminus end and the tryptophan (W) and aspartic acid (D) dipeptide at its C-terminus (hence the name WD40). The region between the GH and WD is conserved and is proposed to coordinate interactions with ligands^22^. EEC11563.1 is a leucine rich repeat (LRR)_8 superfamily member^23^ and EEC14106.1 is a 14-3-3 protein family protein^24^. Both the LRR-8 and 14-3-3 proteins have protein binding properties that could promote tick cement formation. It is interesting to note that in addition to other functions, 14-3-3 protein is an activator of tyrosine hydroxylase^25^, the rate-limiting enzyme that catalyze hydroxylation of tyrosine (Y) leading to synthesis of l-3,4-dihydroxyphenylalanine (l-DOPA). l-DOPA, which has been reported in tick salivary glands but not in tick cement^3^ is important to formation of marine mussel bio adhesives^26^. It is entirely possible that the 14-3-3 tick cement protein here could bind and activate host tyrosine hydroxylase to trigger formation of l-DOPA at the tick feeding site.

### Glycine rich proteins (GRP)

Tick cement has high content of glycine amino acid residues^3^ and on this basis most putative TCPs are GRP^4–7^. In contrast, we identified few GRPs (n = 8) in this study including three (AAY66550.1, EEC14464.1, and EEC00903.1) and five (AAY66821.1, EEC14470.1, EEC15720.1, EEC15718.1, and EEC15723.1) in cement of 6 and 24 h attached ticks. Additionally, CPs (EEC11084.1, EEC11064.1, EEC01924.1, EEC04675.1, XP_02984126.1) have glycine rich domains. The low number of GRPs in this study could be explained by the fact that this study analyzed the inner core layer as opposed to both layers of *A. americanum* cement^4,5^. Some of the GRPs (EEC14470.1, EEC15718.1, EEC15720.1, and EEC15723) and cuticle proteins (CP; EEC11084.1, EEC11064.1, EEC01924.1, and XP_02984126.1) have repeats of the G-G-Y amino acid motifs, which fits the characteristics of the glycine rich family of the CP superfamily^11^. Other GRPs (EEC15708.1, EEC15720.1, and EEC15723.1) have the G-Y-G-G-Y-G-R repeat motif of unknown function. The G residue repeats in the G-G-Y motif are present in structural proteins and is involved in the characteristic triple helices^27^, which contributes to cohesive bonds in extracellular matrices.

### Tick histamine binding proteins (THBP)

We identified four THBPs (AAM93664.1, AAY66819.1, EEC01472.1, and EEC11497.1) in cement of 6 h attached ticks and not in tick mouthparts (ST1A) suggesting specific roles for these proteins in tick cement formation. THBP belong to the lipocalin protein family, which binds several ligands including lipids and biogenic amines such as histamine^28^. Given that lipids are part of tick cement^3^, it is conceivable that putative THBPs in this study might play roles by biding and making lipids available at the tick feeding site. It is also possible that THBPs in this study are part of the tick proteins that mediate tick evasion of the host’s inflammation defense. As histamine is a potent inflammatory agonist^28^, THBPs were postulated and validated to mediate tick evasion of host inflammation defense by sequestering histamine ^28–30^. It is important to note that, although THBPs in this study were identified in cement of 6 h attached ticks only, we have previously shown that both adult and nymph *I. scapularis* ticks secrete THBPs throughout feeding^2,6,7^, demonstrating the importance of these proteins in tick feeding physiology.

### Protease and protease inhibitor (PI).

Although ticks encode for multiple proteases and PIs^31–33^, few were identified in this study. We identified a single thermolysin-like metalloprotease (EEC17055.1) in cement of 6 and 24 h attached ticks and five PIs. The five PIs include three cystatins (cysteine protease inhibitors) in cement of 6 h attached ticks (AAY66685.1, EEC07262.1, and EEC17564.1) and two serine protease inhibitors: Kunitz type (AAM93648.1) and trypsin inhibitor like (EEC18344.1) in cement of 24 h attached ticks (ST1A and ST1C). Thermolysin-like proteases serve as bacteria virulence factors by hydrolyzing extracellular matrix (ECM) proteins such as elastin and keratin to allow bacteria to disseminate^34^. Whether or not deposition of tick cement is preceded by degradation of host ECM proteins has not been demonstrated. However, it is conceivable that the tick degrades host ECM to create space for its feeding site, and the thermolysin-like protease here could serve that role. It is notable that the thermolysin-like protease was identified in cement of 6 h attached ticks, which could suggest its role at start of tick cement formation. There is also evidence that inhibition of cysteine proteases and metalloproteases by broad spectrum synthetic PIs resulted in enhanced deposition of extracellular collagen^35^. Thus, there is potential that cystatins in this study could inhibit host cysteine proteases leading to deposition of extracellular collagen which might play roles in tick cement binding to host skin.

### Cytoskeletal proteins (CSP)

The general property of CSPs to polymerize into structures^36^ suggest their importance to tick cement formation. Putative CSPs in this study include paramyosin (EEC01810.1), muscle myosin (EEC04370.1), and tropomyosin (EEC09701.1) in cement of 24 h attached ticks. Others are actin (AAL30373.1 and EEC03934.1), seven alpha (α) (ADJ68002.1, EEC05710.1, EEC05915.1, EEC12427.1, EEC20577.1, EEC02794.1, and EEC20578.1), and beta (β) (EEC01384.1, EEC03517.1, EEC06076.1, EEC08136.1, EC11984.1, EEC03514.1, and EEC14639.1) tubulins in cement of both 6 and 24 h attached ticks. Native tubulin is a heterodimer of α and β chains^36^, and thus it is likely that seven native αβ tubulins are secreted in tick cement.

Posttranslational modifications (PTMs) that increase functional diversity of αβ tubulin microtubules^37–39^ could promote tick cement formation. Of interest the EEY/F site for αβ tubulin microtubule detyrosination/tyrosination is conserved in seven α-tubulins (ADJ68002.1, EEC05710.1, EEC05915.1, EEC12427.1, and EEC20578.1). In detyrosination, tubulin carboxypeptidase catalyzes removal of the c-terminal tyrosine (Y) and in its place can be replaced by various Y analogues including l-3,4-dihydroxyphenylalanine (l-DOPA). l-DOPA is a post translationally modified Y that plays important roles in bonding of marine mussel glues^26^ but has not been reported in tick cement^3^. It will be interesting to investigate if detyrosination/tyrosination is conserved in tick αβ tubulin and its relationship to binding l-DOPA. Although predominantly protein, lipids and carbohydrates are also part of tick cement^3^ and it is possible that could be respectively added to αβ tubulin through palmitoylation and glycosylation and/or glycation^37–39^. Likewise, αβ tubulin undergoes polyglycylation a process in which a string of glycine amino acids is added and might contribute to formation of triple helices^27^ in tick cement formation.

### Detoxification/antioxidants

Ticks secrete detoxifying/antioxidants in their saliva during feeding to counteract oxidative stress that is triggered by the tick feeding style of disrupting host tissue and then then sucking blood^2,6,7^. Putative detoxifying/antioxidants including NADH-dependent coenzyme Q (CoQ) oxidoreductase (EEC10234.1) and monooxygenase-like (EEC02823.1) in cement of 6 and 24 h attached ticks. Others include putative thioredoxin (AAY66603.1, AAY66786.1, and EEC20239.1), malate dehydrogenase (EEC05916.1), and glutathione peroxidase (EEC00744.1) that were shared by both 6 h and 24 h attached tick cement. On Panther, detoxifying/antioxidants here mapped GO terms related oxidoreductase properties (ST2). In addition to redox functions, oxidoreductases biodegrade numerous biomolecules^40^ and it is conceivable that these proteins could biodegrade host tissue to create space for deposited tick cement and the tick feeding site.

### Enzymes

Putative enzymes in this study include putative lysine-specific histone demethylase (EEC06619.1 and EEC11164.1), inositol polyphosphate-1-phosphatase (EEC02148.1), endonuclease-1 (EEC10745.1), and alpha2-glucosyltransferase (EEC11543.1) in cement of 6 h attached ticks (ST1C). The other enzymes include DNA translocase (EEC05986.1), ecto 5 prime nucleotidase (EEC04477.1), nucleoside hydrolase (EEC17437.1) in cement of 24 h attached ticks, DNA topoisomerase (EEC01195.1), and F0F1-type ATP synthase (EEC17118.1 and EEC02131.1), triosephosphate isomerase (EEC13429.1), nucleoside triphosphate hydrolase (EEC12621.1), and translation initiation factor IF-2 GTPase (EEC12401.1) in both 6 h and 24 h tick cement. Sugars and lipids are part of tick cement^3^, and thus, it is logical to assume that the function of glycosyltransferases to catalyze the transfer of activated sugars to multiple biomolecules including proteins^41^ that could promote tick cement formation.

### Histones

Histones are known for providing structural support to chromosomes^42^. These functions are executed by histone variants that include linker histone H1 and four core histones: H2A (variants: H2A.Z, H2A.B, H2A.X, and macro H2A), H2B, H3 (variants: H3.3 and cenH3), and H4^42^. Here, we identified histone H4 (EEC03614.1) in cement of 24 h attached ticks and in both 6 h and 24 h attached tick cement (EEC09558.1 and EEC09556.1), as well as H2A (EEC09557.1 and EEC06154.1) and H2B (EEC02656.1) in cement of 6 and 24 h attached ticks. Extracellular histones can stimulate collagen expression^43^ a role that might enhance tick cement attachment to host skin.

### Heat shock proteins (HSP)

HSP belong into five families classified by molecular weight (e.g., HSP70 is ~ 70 kDa) including HSP100, HSP90, HSP70, HSP60, and small HSPs^44^. Here we identified mostly HSP70 (n = 8) including six in 24 h attached tick cement (AAT75324.1, EEC01647.1, EEC11639.1, EEC14892.1, EEC19593.1, and EEC20460.1) and two (EEC03688.1 and EEC05056.1) in cement of 6 and 24 h attached ticks. Likewise, the lone putative HSP90 (EEC18473.1) was identified in cement of 6 and 24 h attached ticks. The protein binding properties of HSPs^45^ could contribute to formation of the tick cement complex.

### Tick specific proteins (TSP) of unknown function

TSP were annotated on the basis that they did not show matches to annotated proteins of other organisms and did not have any consensus motifs on the CD database. TSPs were identified in cement of 6 h attached ticks (EEC05763.1, AAY66704.1, AAY66525.1, EEC01412.1, EEC09599.1, EEC03925.1, EEC16392.1), 24 h attached (AAY66555.1, EEC01195.1), and shared (AAY66553.1, AAY66987.1, EEC02397.1, EEC02399.1, EEC03436.1, EEC06365.1, EEC13528.1, AAY66605.1). We did not observe any unique repeat motif with exception of AAY66553.1, AAY66987.1, EEC02397.1, and EEC02399.1 that have c-terminal polyglutamic domains (E-E-E/E-E-K/E-K-K) that have been associated with elastomeric protein properties^46^. Notable in our data is that tick specific protein AAY66605.1 is a homolog of *A. americanum* AV422 protein, which we initially identified among *A. americanum* genes that were up regulated in ticks that were stimulated to start feeding on cattle^47^. We later confirmed that this protein is also highly secreted in saliva by ticks that are stimulated to feed on humans, dogs, and rabbits^48^ and in tick cement that was recovered from mouthparts of partially fed *A. americanum*^4^ and is injected into the host during tick feeding^2,5,6,49^. We have also shown that AV422 is anti-hemostatic and is significant to tick feeding as RNAi-mediated silencing affected tick feeding^50^. It is most likely that this protein regulates other functions in addition to tick cement.

### Miscellaneous function

The miscellaneous function category includes two putative antimicrobial peptides (AMP; AAY66716.1 and EEC20127.1) that were identified in 6 and 24 h attached tick cement. In addition to modulating host immunity, the tick must protect its feeding site from being overrun by secondary bacterial infections, a role that might fulfilled by the two AMPs. We also identified three putative single domain von Willebrand factor type C (VWC) in cement of 24 h attached ticks (AAV80782.1, AAY66767.1, EEC19195.1) that likely have anti-microbial functions^51^. Others identified in cement of 6 h attached ticks include putative Atp11p (EEC20594.1), a ubiquitin-like fold protein (EEC14187.1), and type 3 inositol 1,4,5-trisphospate receptor (EEC19418.1), tick calreticulin (EEC09465.1), and heme lipoprotein (EEC13579.1). Of note, tick calreticulin is one of the well-studied tick saliva proteins and is used as a biomarker for tick bites^52^ and likely involved in tick evasion of the complement defenses^53^. In cement of 24 h attached ticks, we identified a putative vacuolar sorting protein (VPS24; EEC13690.1) that is associated with transport and degradation of both lipids and cellular proteins, 60S ribosomal protein (EEC15712.1), GDP dissociation inhibitor (EEC06563.1) and a transcription factor (EEC16446.1). Those identified in cement of 6 and 24 h attached ticks include putative 40S ribosomal protein S27A (EEC00212.1), ubiquitin/40S ribosomal protein S27A fusion (EEC14178.1), T-complex protein 1 (EEC14604.1).

### In silico physiochemical analysis and putative bond patterns in tick cement

**Figure 5:**
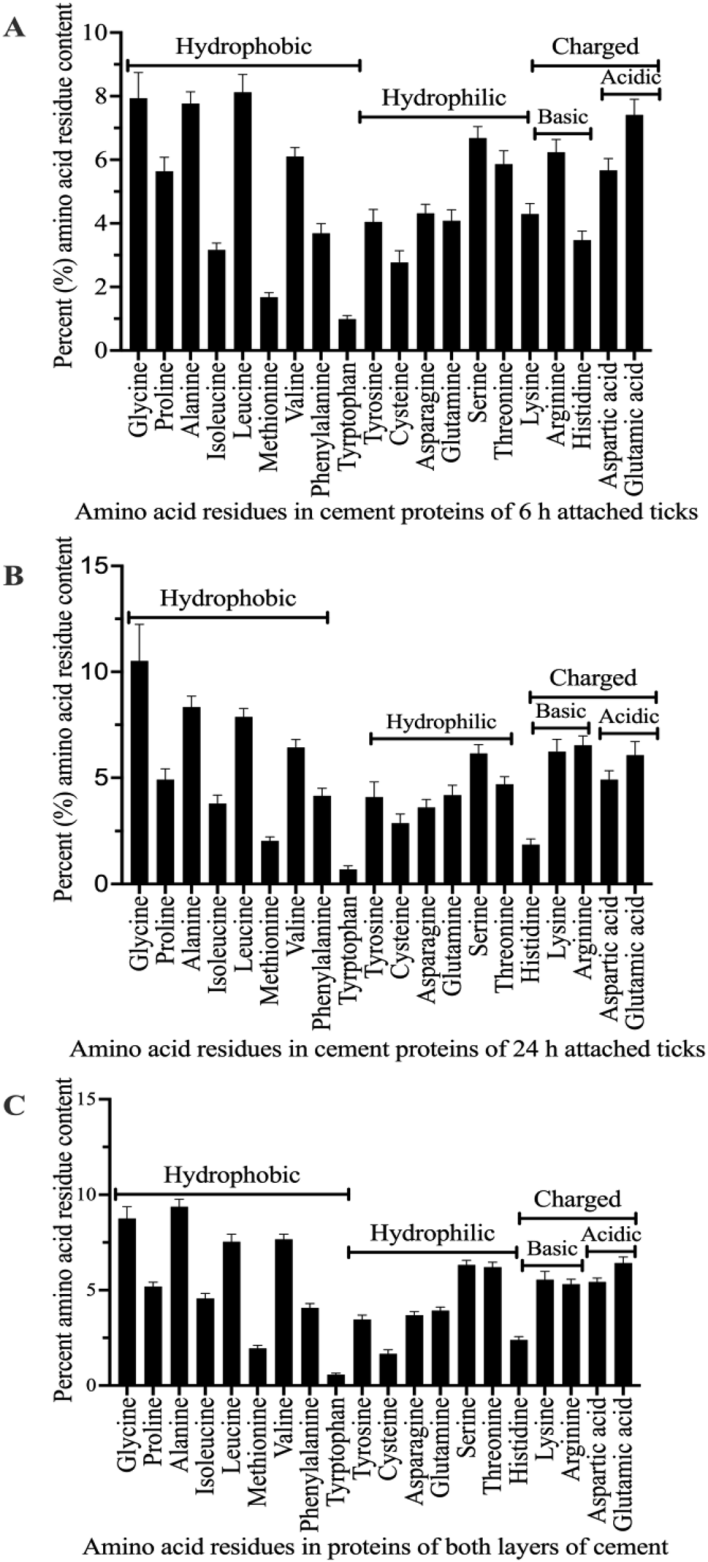
*I. scapularis* tick cement proteins have high content of hydrophobic amino acid residues. Counts of amino acid residues were enumerated on bioinformatics.org server (https://www.bioinformatics.org). Amino acid residue counts were plotted in PRISM 9. Classification of amino acid residues as hydrophobic and hydrophilic reflect likelihood to interact with water while charged (basic and acidic) being charged at neutral pH as defined in^10^.

To gain insights into physiochemical properties, we profiled amino acid residues (Fig. 5). As shown (Fig. 5), *I. scapularis* tick cement proteins have high content of hydrophobic residues, followed by hydrophilic and basic amino acid residues. Data here is similar to chemical analyses findings of high content of hydrophobic residues in tick cement^3,4,54^. The amino acid residue composition in tick cement proteins is comparable to major extracellular matrix (ECM) in the skin. For instance, collagen the main ECM protein in the skin has high content of hydrophobic amino acids, G, P, and A^55^. This similarity might explain the biocompatibility of tick cement and host skin. Post translational modifications (PTMs), glycosylation, phosphorylation, and hydroxylation promote bio adhesive bond formation in marine animal bio adhesives^56–58^. It is interesting to note that PTMs are predicted in some of the TCPs (ST1C). Importantly, some of the enzymes identified here catalyze PTMs relevant to tick cement formation. Notably, monooxygenase (EEC02823.1) in cement of 6 and 24 h attached ticks (ST1A), flavin containing monooxygenase (EEC02063.1) and 4-hydroxyphenylpyruvate dioxygenase (EEC06118.1) in tick mouthparts (ST1B) will catalyze hydroxylation^59^. Likewise, alpha2-glucosyltransferase (EEC11543.1) in cement of 6 h attached ticks (ST1A) is associated with glycosylation of proteins^60^. Interestingly, putative 14-3-3 epsilon (EEC14106.1) in cement of 6 and 24 h attached ticks is an activator of tyrosine hydroxylase, which catalyzes hydroxylation of tyrosine leading to synthesis of l-DOPA^25^, which promote bond formation in marine mussel^26^. Interestingly, we observed variants of rabbit tyrosine 3-monooxygenase/tryptophan 5-monooxygenase activation protein eta (YWHAH)^61^ among rabbit proteins that were associated with tick cement of (ST1G). Therefore, it is entirely possible that the 14-3-3 tick cement protein or the tyrosine 3-monooxygenase enzyme from either ticks or rabbits to trigger formation of l-DOPA at the tick feeding site. We recognize the fact that PTMs are intracellular pathways and whether they occur at the tick feeding site remains to be validated. Disulfide bonds hold together secondary structures^62^ that will be critical to cement formation. Thus, it is exciting to note that 107 of the 138 TCPs in this study have at least two cysteine residues (ST1C) indicating the possibility some these proteins have functional disulfide bonds and likely involved in cement formation.

## Conclusions and future perspectives

This study for the first describes proteins in the inner core layer of *I. scapularis* tick cement. The inner core tick cement layer initiates attachment of tick mouthparts to host skin and serves as the anchor for the outer cortical cement layer, which stabilizes the tick feeding site^3^. We speculate that targeting some of the TCPs in this study will affect deposition of the outer cortical tick cement layer and destabilize the tick feeding site. We have previously showed that disruption of tick cement is tenable in that RNAi-mediated silencing of selected transcripts encoding for *A. americanum* cement proteins significantly affected the integrity of the tick attachment site^4,63^. Since tick cement is deposited into host skin, it is considered well adapted to function in a live host with limited side effects. From this perspective, data here could lead to synthesis of medical bio adhesives that are well tolerated by mammalian skin.

The design of this study was not set out to determine the host response to tick cement deposition as the non-infested rabbit skin was not analyzed and some of the rabbit proteins might be dismissed as contamination. However, inspection of rabbit proteins associated with *I. scapularis* cement (ST1G) reveals the possibility of rabbit response to deposited tick cement. For instance, rabbit antimicrobial peptides and blood clotting system factors were associated with cement of both 6 and 24 h fed ticks (ST1G). In addition to suppression of host innate immunity, the tick must prevent secondary colonization of its tick feeding site by bacteria in the skin, and thus the rabbit microbial peptides associated with tick cement could serve this role. Likewise, blood clotting system factors likely represent the host response to tissue injury caused by tick mouthpart as it penetrates host skin to start feeding. While descriptive, data here provide the foundation for to understand the molecular basis of tick cement formation, design anti-tick vaccine antigens to disrupt tick cement formation, and synthesis of medically beneficial bioadhesives.

## Supplementary Information

Supplementary Figure 1.

Supplementary Figure 2.

Supplementary Table 1.

Supplementary Table 2.

## Data Availability

The mass spectrometry proteomics data and the database that was used for this analysis have been deposited to the ProteomXchange Consortium via the PRIDE partner repository (Accession# PXD038685). Please note that the GenBank accession numbers for tick proteins and Uniprot accession numbers for rabbit proteins are included in Supplemental Table 1.
