# Supplementary material for: Identification and characterization of proteins that form the inner core Ixodes scapularis tick attachment cement layer
Source: Sci Rep. 2022 Dec 9;12:21300. doi: 10.1038/s41598-022-24881-4 (PMC9734129; doi:10.1038/s41598-022-24881-4)
Supplement: Supplementary file 1 — Supplementary Figure 1. [file 41598_2022_24881_MOESM1_ESM.pptx]

## Slide 1
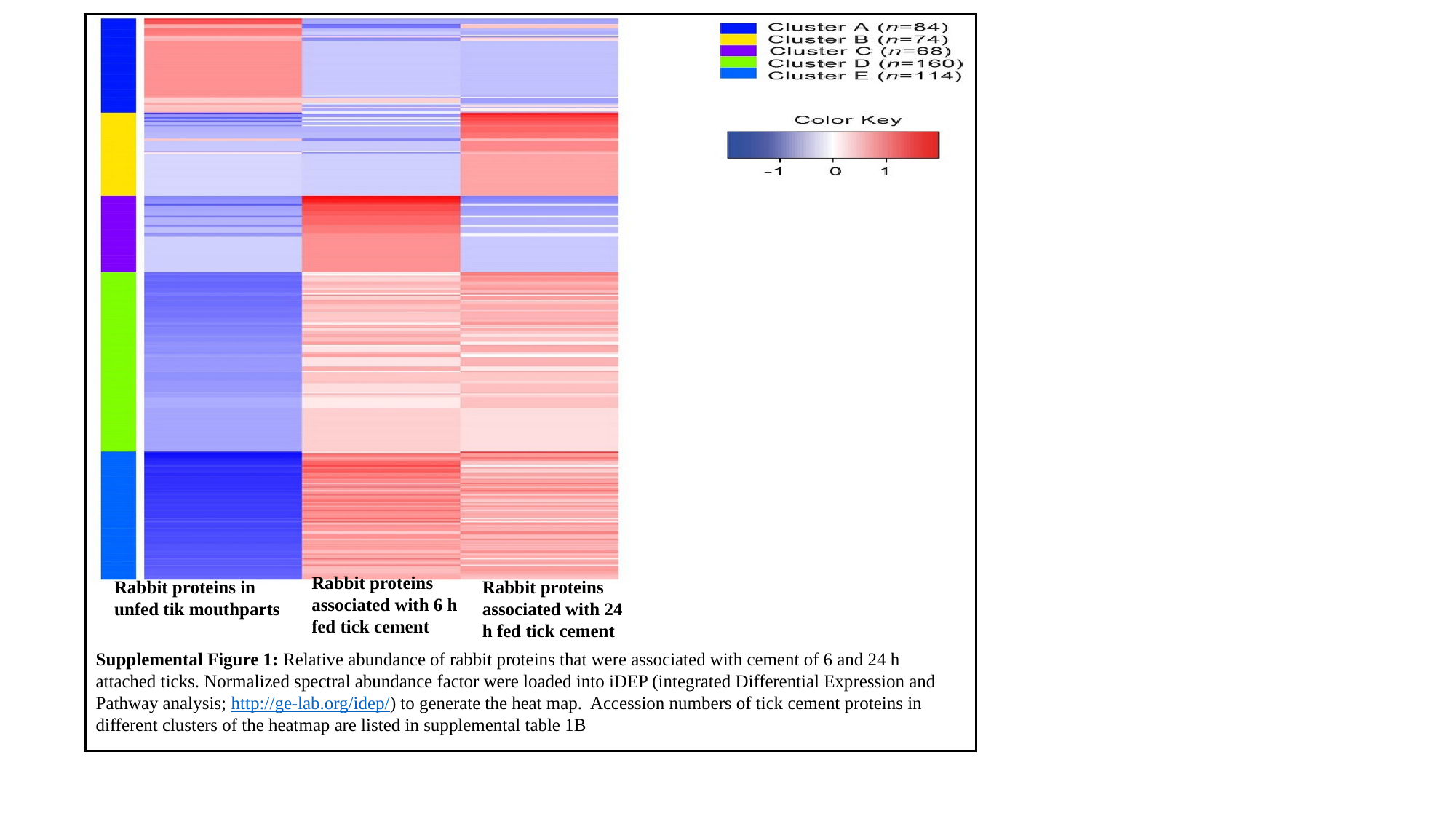

Rabbit proteins associated with 6 h fed tick cement
Rabbit proteins in
unfed tik mouthparts
Rabbit proteins associated with 24 h fed tick cement
Supplemental Figure 1: Relative abundance of rabbit proteins that were associated with cement of 6 and 24 h attached ticks. Normalized spectral abundance factor were loaded into iDEP (integrated Differential Expression and Pathway analysis; http://ge-lab.org/idep/) to generate the heat map. Accession numbers of tick cement proteins in different clusters of the heatmap are listed in supplemental table 1B
