# Supplementary material for: Identification and characterization of proteins that form the inner core Ixodes scapularis tick attachment cement layer
Source: Sci Rep. 2022 Dec 9;12:21300. doi: 10.1038/s41598-022-24881-4 (PMC9734129; doi:10.1038/s41598-022-24881-4)
Supplement: Supplementary file 2 — Supplementary Figure 2. [file 41598_2022_24881_MOESM2_ESM.docx]

**Supplemental Fig.2A**

>XP_029841824.1 uncharacterized protein LOC8051553 [Ixodes scapularis]

MFQIALLLACVCVTVAQRGFGGAGGEVYAPIP**YAFNYLSQLEDGSHSHEEQGDGNGRVSGKYTLTTSDGRTRTVTYTADENGF**RAQVDTNEQGTESKNPADVVFQSSALPGPEAAIANEPNRVRGGPAPGGFRVLGNVGQRPLIGRRYPAGSRPSDYKDERRQKASTSSQQHLLHWLYSFLVSRERATRHHRFNMHKIALFMACVCAAAAQRFGPDENYAPIP**YAFNYLSQLVDGSHSHEEQGDGNGRVTGRYTLTDADGRSRTVTYTADENGF**RAQVETNEQGTESKNPADVVFQSSALPGPEAAIANEPNRFRGAAAPRVVMRPIYFSPLGYMLCSPEEENLNLVRIPLFLMFQVALLLACACVVAAQRFGVARQDEFYGPPIP**YAFNYLSQLVDGSHSHEENGDGNGRVTGRYTLTDADGRTRTVTYTADENGF**RAEVVTNELGTESKNPADVVFRSSAPTGPEAAIANEGNRLRAGPVFFIDIFQITLLLACISVAAAQRGFQENYGPPIP**YAFNYISQLADGSHTHEQSRDGSGRVTGKYTLSDADGRTRTVTYYADDTGF**HADVVTNEQGTESKNPADVTIKSSALPGPEAAIANEPNRIRGGPAAAGFRGQRSLIGRHRPQRARWNLYKTEAAQRGFEENYGPPIP**YAFNYLSQLQDGSHSHEQSGDGSGRVQGKYTLTDADGRSRTVTYYADASGF**HAEVITNELGTESKNPADVVIRSSAPTGPEAAIANEPNRFREGALTSEIFTLFESKRHAAAQRGLEENYGPPIP**YAFNYLSQLEDGSHSHEQSGDSNGRVQGKYTLTDADGRTRTVTYYADETGF**HADVVTNELGTESKNPADVTIQSSAPTGAEAAFANPGPAAGGFRG

**Supplemental Fig.2B**

>EEC11084.1 hypothetical protein IscW_ISCW007105 [Ixodes scapularis]

MIAQVTLVLGLVAVALGGGHHGGHSQTYRNQDDHGH**YRFGYDIANGYGAVNGRHEKGAAYGPVHGSYYLGDVDGRHRRVEYVADKLGF**RAVVKTNEPGTKTSLPAAAPYISVICGLVAVATAGVVGPYGGFSRQHRKQDDFGR**YNFGYNIVNGLGATNSRYESGSAYGPVVGSYTLADIDGRARRVEYVADKLGF**RAVVKTNEPGTKTSLAAPHPTSRPRDPCYVLKDSDDYGYFLPNGTGTGTMRMLQEKETDALPIPFGLDLDMFTQFHAMPLVRNTDLHIMTGTMKPFVDNAFAIAIGFDPVMGILIAVTLLTLTLLTVLLEGTGITSWFSRVLSYWAQLFMEPSSVLPSKPSTRALFGAWLLGCLVVNSWFSAVICGSLLVQVPSKRIDTADDLLNATHIRPTSIDNIPLLAVMRMSTKPSVRRVYWKMQELRGQLPVETVYSLDYLRAMQEQKHSLMIDMTSMSFVEERTCPHLKGFFYFGEEIIDVLRMTWYTRKDLPPDVLQTMEERTTRIIESGLEFMRPQAHTARQYTCTLNTLPKGNSVRYDPLTLKDIHFISIICGFWLMAAAIALDYGQ**YEFGYDIVNGHGAVNGRHETGSAHGPVVGSYYLGNIDGRHRRVSYVADKLGF**RAVVKTNEPGTKSSYPAASATISANGKYVPSAGFSHGGYGGGGYGGGGGYGGGGHGLSGHESVGYVVGYPLLLLFRAPP

**Supplemental Fig.2C**

>XP_029841826.1 glycine-rich cell wall structural protein [Ixodes scapularis]

MWKLTISTLCFAALVNAGNLRGGGGFGGGGFGGGGFGGAGHHGGGGFGGGGLGGGHHGGGGFGGGGLGGGGLGGGGLGGGFGGHHGGGLGGGLGGGFGGGHHGGGGFGGGGRGGYGGGFSSGLGGGYRGGGGGYGGGGYGGGRYVDESPKP**YQFGYDAQGPDGSSSRQETSDGSGRVQGSYTITTADGLQRKVKYAADEGGF**RARVDTNEPGTTNDSPADVTLQSSAPSPAELSAQYTASRGYGRGGGGYGGGGYGGAGAGYGGGRFGGGYGGGHHGGYGGGGYGGGRGGFGGGLGGGAGGLGGLGGGHGGFGGGAGGFGRGGGGGGFGGPAGGGVTVSAFKVHHGGGGAGGYGGLGGGGGYRGGGGGGYGGAGGFGGSGGYGGSGGFGGAGGYGGAGGFGGGKYGGGAGGAGGGGGWKW

**Supplemental Fig.2D (pfam01607)**

>EEC12627.1 conserved hypothetical protein [Ixodes scapularis]

MSLEKALAQPRVPSWREAADDLKTRGRRPRISPEMRRHALLLPGKGETRAAPAKEEVYKGQVYFARIVFSPLVRRLLHSFLPPCVWFCFFWGPSSAVLCLLLLVACHPRGLPAPPSPAHLESHLPRSGPDGAANMRLTILAIAVIATLATASAYKPKRQVQYQPGGSRGSSAAPYEGYQQQQPQAQSFRPQVSAPPQQGYDPNVYQPQQQAQPRRQTVQSFGGGLARPQQQQQRPQQQDNRQQTQSEEEEEEQKPNPLTLLLEKSTFT**CSGKTDGYYSDNSVDCQVFHYCVAGAKHSWMCPEGTVFHQVHLNCVPASQ**DICNTAEKFFFVNDYLHKELESRGPNNTVQYAQRYYPDGYVLGDPFTVPSGSQPQPQAPQQQYQEPRQQPQPQPRQRFRPEQAPRGPPAGFQQYQPQPPSYTAQAPSQQPASSPAQFGGAQPSRGSGNFGGLPPTRVYTIPAQARPAADATPSYLYRQGGSPSTAQQQQQSSVKTSTTSRSSIVLPPSSPKTGNFTSTKLPPHPG

﻿ **Supplemental Figure 2:** Tick cement cuticle proteins with multiple chitin/carbohydrate binding domains and other amino acid motif repeats of unknown function. Chitin (pfam00379) in SF1A-1C and carbohydrate binding in SF1D are bolded. Repeat motifs (in all sequences) and glycine rich domains in SF1C and 1D are underlined and shaded in dark and light gray respectively.
